# Lung functions among patients with pulmonary tuberculosis in Dar es Salaam – a cross-sectional study

**DOI:** 10.1186/s12890-016-0213-5

**Published:** 2016-04-23

**Authors:** Mohamed Manji, Grace Shayo, Simon Mamuya, Rose Mpembeni, Ahmed Jusabani, Ferdinand Mugusi

**Affiliations:** Department of Internal Medicine, Muhimbili University of Health and Allied Sciences (MUHAS), P.O. Box 65001, Dar es Salaam, Tanzania; Department of Environmental Occupational Health, Muhimbili University of Health and Allied Sciences (MUHAS), P.O. Box 65001, Dar es Salaam, Tanzania; Department of Epidemiology and Biostatistics, Muhimbili University of Health and Allied Sciences (MUHAS), P.O. Box 65001, Dar es Salaam, Tanzania; Department of Radiology, The Aga Khan Hospital, P.O Box 2289, Dar es Salaam, Tanzania

**Keywords:** Spirometry, TB sequlae, Obstruction, Restriction

## Abstract

**Background:**

Approximately 40–60 % of patients remain sufferers of sequela of obstructive, restrictive or mixed patterns of lung disease despite treatment for pulmonary tuberculosis (PTB). The prevalence of these abnormalities in Tanzania remains unknown.

**Methods:**

A descriptive cross-sectional study was carried out among 501 patients with PTB who had completed at least 20 weeks of treatment. These underwent spirometry and their lung functions were classified as normal or abnormal (obstructive, restrictive or mixed). Logistic regression models were used to explore factors associated with abnormal lung functions.

**Results:**

Abnormal lung functions were present in 371 (74 %) patients. There were 210 (42 %) patients with obstructive, 65 (13 %) patients with restrictive and 96 (19 %) patients with mixed patterns respectively. Significant factors associated with abnormal lung functions included recurrent PTB (Adj OR 2.8, CI 1.274 - 6.106), Human Immunodeficiency Virus (HIV) negative status (Adj OR 1.7, CI 1.055 - 2.583), age more than 40 years (Adj OR 1.7, CI 1.080 - 2.804) and male sex (Adj OR 1.7, CI 1.123 - 2.614).

**Conclusion:**

The prevalence of abnormal lung functions is high and it is associated with male sex, age older than 40 years, recurrent PTB and HIV negative status.

## Background

Tuberculosis (TB) is among the top infectious causes of death worldwide [1]. Patients with treated TB may remain lifelong sufferers of disabling sequelae of the disease which subsequently impair their quality of life [2]. Many studies have shown that partially treated pulmonary tuberculosis can result in airflow obstruction [3–7]. Studies with longer follow-up have revealed that a large percentage of patients with treated pulmonary tuberculosis show signs of permanent airflow obstruction or restrictive impairment [8–11]. Post tuberculosis pulmonary impairment, therefore has emerged as a distinct clinical entity [12].

With 170,000 reported TB cases in the year 2014, Tanzania ranked the 18^th^ among the 22 WHO high TB burdened countries in the world [13]. The prevalence of bacteriologically confirmed TB in Tanzania was 295 per 100,000 adults in the year 2010 [14]. Despite this high burden of TB, there is no formal evidence of post tuberculous pulmonary abnormalities from Tanzania. Factors associated with these complications or factors that may predict long term residual abnormalities in such patients has also been poorly addressed. This study was aimed at determining the prevalence and the factors associated with these abnormalities.

## Methods

### Study design and population

We conducted a health facility-based descriptive cross sectional study between July and December 2014 in Temeke municipality, the largest of three municipalities in Dar es Salaam city. Two busy TB clinics in this municipality, the Temeke municipal hospital and the Mbagala rangi tatu health centre, were chosen. The target population was patients with pulmonary tuberculosis (PTB), both sputum smear positive and smear negative TB regardless of their HIV status. Eligible patients were aged 18 years or older, had attended the clinic and completed at least 20 of the 24 weeks of anti TB treatment. Prior research has shown that peak lung function loss in patients with TB occurs within 6 months of diagnosis [8]. We enrolled patients who had completed 20 of the 24 weeks of anti TB treatment (i.e. 5 months), since most of these subjects would have achieved a microbiological cure, and that such timing would enable identification of sequel of disease without considering active disease. This timing was also reasonable in allowing data collection for a maximal number of subjects given that patients are discharged from the clinic at 6 months after which it would be difficult to access them. Participants were enrolled in a consecutive manner. The exclusion criteria were participants who had preexisting asthma or COPD, paraplegia, heart failure, stroke, extra pulmonary tuberculosis, contraindications to spirometry (e.g. recent surgery, myocardial infarction, vertigo, vomiting), spine deformities (kyphosis, scoliosis), chest deformities, and pregnancy.

**Fig. 1:**
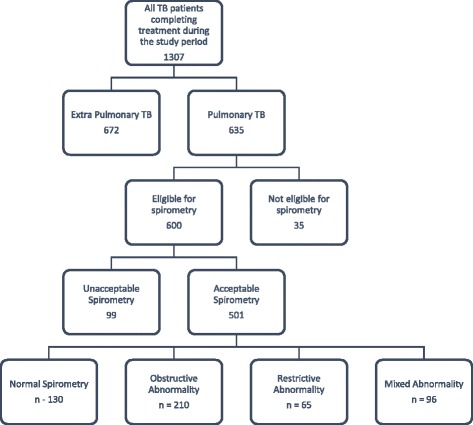
Flow diagram for recruitment into the study

A total of 501 participants were enrolled in the study (Fig. 1). All participants gave consent for participation. Ethical clearance for conducting the study was obtained from the Muhimbili University of Health and Allied Sciences (MUHAS) institutional review board.

### Data collection

Evaluations were incorporated into routine TB clinic visits. A clinical record form (CRF) was used for data collection. A thorough history and physical examination was performed for each participant and recorded on the CRF. The modified Medical research council dyspnea scale was used to assess their functional status [15]. The patients TB02 treatment card was used to obtain other relevant information including duration on anti TB treatment, serostatus for HIV and whether the PTB was smear negative or positive for acid fast bacilli (AFB) at diagnosis. This information was filled in the CRF. A digital weighing scale (OMRON) was used to measure body weight in kilograms (kg). Weight was measured in erect position wearing only light clothing and without shoes. Height was measured in centimeters using meter rods. Waist circumference was measured using a tape measure midway between the lower rib cage margin and the anterior superior iliac spine. Mid upper arm circumference (MUAC) was measured with a tape measure midway between the tip of the shoulder (the acromion) and the tip of the elbow (olecranon process).

This was followed by spirometry by means of the ndd EasyOne™ spirometer (manufactured by ndd Medizintechnik (Switzerland)). The EasyOne™ spirometer complies with the 2005 ATS/ERS spirometry standards. The spirometer does not need daily calibration [16–18]. It has been used in several studies before both locally and internationally [19–22]. Thus pre testing of the tool was not performed. The device is portable and not affected by environmental conditions. Precautionary measures in using the spirometer included the use of disposable mouthpiece (spirette) per participant to avoid cross contamination. Spirometry was performed in a vacant area with open sunlight and face masks and gloves were used to protect the investigator/assistants.

The forced expiratory maneuvers, the within and between maneuver evaluation for acceptability as well as test result selection were in accordance with the ATS/ERS guidelines [23]. Spirometry was performed in the standing position. The forced expiratory maneuvers were explained in local language by trained assistants (3 doctors and 1 nurse) to the participants before they underwent spirometry. Spirometry was performed without a nose clip owing to the results of a pilot study among 25 patients prior to this research that observed that patients were uncomfortable when nose clips were used during spirometry. Furthermore, a nose clip is recommended, but not a compulsory requirement for spirometry [23] and other studies have not shown significant differences in results among users and non-users of nose clips [24, 25]. Disposable mouthpieces (spirettes) were discarded after individual use. Parameters measured included forced expiratory volume in one second (FEV1) and the forced vital capacity (FVC). The highest FEV1 and FVC from three acceptable maneuvers were used for further comparison and analysis. The FEV1: FVC% was then calculated from these.

### Data analysis

The lung functions were categorized into normal, obstruction, restriction and mixed patterns of lung disease according to the algorithm from the National Lung Health Education Program (NLHEP) [26].

The predicted values were derived from regression equations for healthy Tanzanian adults [22]. To compare the observed and predicted lung functions, we used the percentage of predicted values (the ratio of observed to predicted values times 100) for FVC (FVC %) and FEV1 (FEV1%). Severity for obstruction and restriction were graded according to Global Initiative for Chronic Obstructive Lung Disease (GOLD) [27] and the American Thoracic Society/European Respiratory Society (ATS/ERS) task force recommendations [28] respectively.

Data was analyzed using SPSS (version 15.0) statistical software. Means ± standard deviation (SD) and proportions were calculated for continuous and categorical data respectively. Median and interquartile range (IQR) was used for non-normally distributed numerical data. The Chi square test and t-test were used to determine associations between variables. If more than 20 % of the cells had an expected frequency of <5, Fischer’s exact test was used instead.

Logistic regression was done to determine predictors of abnormal lung function. The dependent variable was lung function which was reported as normal or abnormal. The independent variables that were run in multivariate models were those that had a p value of < 0.2 in univariate analysis. These included age, sex, level of education, Modified Medical Research Council Dyspnea Score (MMRC Score), smoking status, sputum smear status at diagnosis (positive or negative), TB episode number (first episode or recurrent), duration from symptoms to diagnosis of TB, HIV serostatus (positive or negative) and CD4 counts. Statistically significance was achieved when *p* < 0.05.

## Results

### Study population

There were a total of 501 participants in the study (Fig. 1). Males constituted 60.5 % (303/501) of the study participants. More than 60 % (326/501) of participants were aged 40 years or younger. Majority were married or cohabiting (52.5 %) (263/501), had attained primary level education (65.9 %) (330/501) and were domestic workers (34.1 %) (171/501). Approximately 29.3 % (147/501) were ever smokers with a median of 2.5 pack years. With respect to their clinical characteristics, approximately 54.3 % (272/501) had smear positive pulmonary TB and 90.1 % (245/272) of these had converted to smear negative at 2 months of treatment. Majority (85 %) (426/501) of the patients had presented with a first episode of TB. The median time from first symptom presentation to TB diagnosis was 30 days. Approximately 30 % (152/501) of the population was HIV co-infected. Approximately 46 % (70/152) of the patients had CD4+ cell count <200cells/μL. Majority (81 %) (407/501) had good functional scores (1 to 2) for dyspnea as reported by the MMRC score (Table 1). The mean FEV1, FVC and FEV1: FVC% were 1.83 ± 0.75, 2.95 ± 1.11 and 63.02 ± 17.99 respectively.

**Table 1:** Sociodemographic and clinical characteristics of participants

Characteristics (N = 501)	Frequency	Percent (%)
Age Groups (years)		
18–30	166	33.1
31–40	160	31.9
41–50	96	19.2
51–60	42	8.4
>60	37	7.4
Sex		
Male	303	60.5
Female	198	39.5
Marital Status		
Single	169	33.7
Married/Cohabiting	263	52.5
Divorced/Widowed	69	13.8
Education		
No Formal	62	12.4
Primary	330	65.9
Secondary	84	16.8
Post-Secondary	25	5.0
Occupation		
Agriculture	19	3.8
Domestic	171	34.1
Industrial	64	12.8
Office	119	23.8
Other	128	25.5
MMRC score		
1–2	407	81.2
3–5	94	18.8
Cigarette smoking		
Never smokers	354	70.7
Ever Smokers	147	29.3
Smoking Pack Years (Median (IQR))	2.5 (5.3)	
Smear for Acid Fast Bacilli		
Positive	272	54.3
Negative	229	45.7
Time to Negative Sputum Conversion (months)(n=272)		
Smear Negative at 2 Months	245	90.1
Smear Positive at 2 Months	27	9.9
TB Episode		
First	426	85.0
Recurrent	75	15.0
Duration to TB diagnosis (months)		
≤1 Month	275	54.9
>1 Month	226	45.1
HIV Status		
Positive	152	30.3
Negative	349	69.7
CD4 count (n = 152)		
<200	70	46.2
200–350	50	33.0
>350	32	20.9

*MMRC Score* Modified Medical Research Council Dyspnea Score, *BMI* Body Mass Index, *IQR* Interquartile range

### Prevalence and severity of abnormal lung functions

**Fig. 2:**
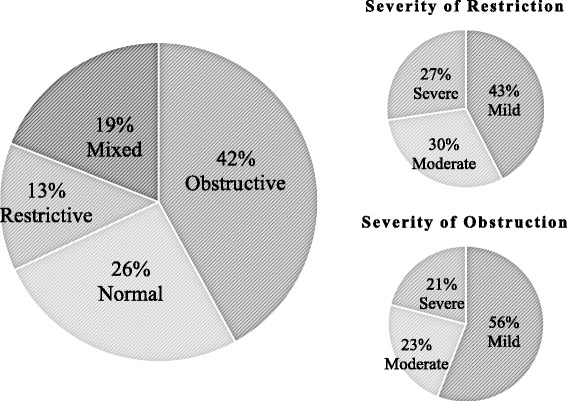
Prevalence and severity of abnormal lung functions. Pie charts showing the prevalence of the different types of lung function abnormalities (large pie chart, *N* = 501). The smaller pie charts show the severity of patients who had restrictive (small upper, *n* = 65) and obstructive abnormalities (small lower, *n* = 210)

The overall prevalence of lung function abnormalities was 74 % (371/501). Majority was due to obstruction (42 %) (210/501) followed by mixed (19 %) (96/501) and restrictive (13 %) (65/501) abnormalities. Approximately 73 % (48/65) of patients with restrictive dysfunction has mild to moderate severity. Approximately 79 % (166/210) of patients with obstructive dysfunction had mild to moderate severity (Fig. 2).

### Factors associated with abnormal lung functions

Patients who were aged 40 years or younger were less likely to present with abnormal lung functions (69.9 %) compared to those older than 40 years (81.7 %), *p* = 0.004. Abnormal lung function was more prevalent among males (78.9 %) than among females (66.7 %) (*p* = 0.002). However more males developed restrictive (*p* = 0.034) and mixed (*p* < 0.001) abnormalities while more females developed obstruction (*p* < 0.001) (Table 2). As far as clinical characteristics are concerned, higher MMRC scores were significantly associated with any abnormality (84 % for scores 3–5 vs 71.7 % for score 1–2) (*p* = 0.013). A similar finding was seen for mixed abnormalities (*p* = 0.009). However lower scores were significantly more associated with obstruction (*p* = 0.04). Patients who had more than 5 pack years of smoking had significantly higher obstructive abnormalities than those who had smoked for less than 5 pack years (73.3 % vs. 41.9 %) (*p* =0.005). Patients with recurrent TB were also more likely to have abnormal lung functions compared to those with a first episode of TB (89.3 % vs 71.4 %) (*p* = 0.001). Also HIV negative patients constituted significantly greater percentage of individuals with abnormal lung functions (77.1 %) compared to HIV infected patients (67.1 %) (*p* = 0.019). This relationship was also true for mixed lung disease (*p* = 0.012). However obstructive dysfunction was more common in HIV infected (66.7 %) than in HIV negative patients (52.4 %) (*p* = 0.014) (Table 3).

**Table 2:** Sociodemographic factors associated with abnormal lung functions

Characteristics	Total	Any Abnormality		*P*	Obstructive		*P*	Restrictive		*P*	Mixed		*P*
Age groups (number, %)													
≤40 years	326	228	69.9 %	0.004*	121	53.1 %	0.132*	46	20.2 %	0.163*	61	26.8 %	0.715*
>40 years	175	143	81.7 %		88	61.5 %		20	14.0 %		35	24.5 %	
Sex (number, %)													
Males	303	239	78.9 %	0.003*	112	46.9 %	<0.001*	50	20.9 %	0.034*	77	32.2 %	<0.001*
Female	198	132	66.7 %		97	73.5 %		16	12.1 %		19	14.4 %	
Marital Status (number, %)													
Single	169	120	71.0 %	0.432	64	53.3 %	0.679	23	19.2 %	0.723	33	27.5 %	0.874
Married/Cohabiting	263	201	76.4 %		115	57.2 %		36	17.9 %		50	24.9 %	
Divorced/W idowed	69	50	72.5 %		30	60.0 %		7	14.0 %		13	26.0 %	
Education (number, %)													
No Formal Education	62	47	75.8 %	0.080	29	61.7 %	0.063	7	14.9 %	0.912	11	23.4 %	0.036
Primary Education	330	249	75.5 %		148	59.4 %		45	18.1 %		56	22.5 %	
Secondary Education	84	62	73.8 %		26	41.9 %		11	17.7 %		25	40.3 %	
Post-Secondary Education	25	13	52.0 %		6	46.2 %		3	23.1 %		4	30.8 %	
Occupation (number, %)													
Agriculture	19	18	94.7 %	0.05	11	61.1 %	0.005	1	5.6 %	0.236	6	33.3 %	0.013
Domestic	171	119	69.6 %		81	68.1 %		19	16.0 %		19	16.0 %	
Industrial	64	44	68.8 %		16	36.4 %		10	22.7 %		18	40.9 %	
Office	119	96	80.7 %		53	55.2 %		14	14.6 %		29	30.2 %	
Other	128	94	73.4 %		48	51.1 %		22	23.4 %		24	25.5 %	

* fishers exact

**Table 3:** Clinical factors associated with abnormal lung functions

Characteristics	Total	Any Abnormality		*P*	Obstructive		*P*	Restrictive		*P*		Mixed	*P*
MMRC score (number, %)													
1–2	407	292	71.7 %	0.013*	173	59.2 %	0.04*	53	18.2 %	0.868*	66	22.6 %	0.00
3–5	94	79	84 %		36	45.6 %		13	16.5 %		30	38.0 %	9*
Cigarette smoking (number, %)													
Never smokers	354	255	72.0 %	0.118*	151	59.2 %	0.114*	49	19.2 %	0.309*	55	21.6 %	0.00
Ever Smokers	147	116	78.9 %		58	50.0 %		17	14.7 %		41	35.3 %	7*
Smoking Pack Years (number, %) (n=147)													
≤5	107	86	80.4 %	0.5*	36	41.9 %	0.005*	16	18.6 %	0.068*	34	39.5 %	0.12
>5	40	30	75.0 %		22	73.3 %		1	3.3 %		7	23.3 %	6*
Smear for Acid Fast Bacilli (number, %)													
Smear Positive	272	198	72.8 %	0.54*	110	55.6 %	0.754*	31	15.7 %	0.278*	57	28.8 %	0.19
Smear Negative	229	173	75.5 %		99	57.2 %		35	20.2 %		39	22.5 %	2*
Time to Negative Sputum Conversion (number, %)													
Smear Positive at 2 Months	27	19	70.4 %	0.82*	11	57.9 %	1.0*	3	15.8 %	1.0*		26.3 %	1.0*
Smear Negative at 2 Months	245	179	73.1 %		99	55.3 %		28	15.6 %			29.1 %	
TB Episode Number (number, %)													
First	426	304	71.4 %	0.001*	171	56.3 %	1.0*	59	19.4 %	0.111*	74	24.3 %	0.16
Recurrent	75	67	89.3 %		38	56.7 %		7	10.4 %		22	32.8 %	6*
Duration to TB diagnosis (mo) (number, %)													
≤1 Month	275	207	75.3	0.539*	117	56.5 %	1.0*	40	19.3 %	0.414*	50	24.2 %	0.40
>1 Month	226	164	72.6		92	56.1 %		26	15.9 %		46	28.0 %	6*
HIV Status (number, %)													
HIV Positive	152	102	67.1 %	0.026*	68	66.7 %	0.014*	17	16.7 %	0.764*	17	16.7 %	0.01
HIV Negative	349	269	77.1 %		141	52.4 %		49	18.2 %		79	29.4 %	2*
CD4 count (Median (IQR))		202.50 (210)				192.5 (225)			200 (156)			260(167)	

* fishers exact, *MMRC score* Modified Medical Research Council Dyspnea score, *BMI* Body Mass Index, *IQR* Interquartile range

In multivariate analysis significant predictors for any abnormality were age > 40 years (Adj OR 1.740, CI 1.080 - 2.804), male sex (Adj OR 1.713, CI 1.123 - 2.614), TB recurence (Adj OR 2.789, CI 1.274 - 6.106) and HIV negative status (Adj OR 1.651, CI 1.055 - 2.583) (Table 4).

**Table 4:** Logistic regression analysis of sociodemographic and clinical parameters

Characteristic (*n* = 501)	Comparison	OR	95 % CI	*P*	Adj OR	95 % CI	*P*
Age >40 years	≤40 years	1.921	1.224–3.014	0.005	1.740	1.080–2.804	0.023
Male	Female	1.867	1.24 -2.797	0.002	1.713	1.123–2.614	0.013
Any Formal Education	No Formal	0.899	0.944–8.087	0.736	-	-	-
MMRC score 3–5	MMRC ≤2	2.074	1.147 -3.752	0.016	1.786	0.962–3.316	0.066
Ever Smoker	Never smoker	1.453	0.918–2.300	0.111	-	-	-
Smear Positive TB	Smear Negative	0.866	0.579–1.296	0.484	-	-	-
Smear Positive at 2 months	Smear negative at 2 months	0.876	.366–2.096	0.766	-	-	-
TB recurrence (Any recurrence)	1^st^ episode	3.361	1.568–7.206	0.002	2.789	1.274–6.106	0.010
Duration to Diagnosis >1 month	≤1 months	0.869	0.582–1.297	0.492	-	-	-
HIV Negative	HIV Positive	1.648	1.082–2.510	0.020	1.651	1.055–2.583	0.028
CD4 count >350	<350	0.362	0.124–1.059	0.064	-	-	-

*MMRC Score* Modified Medical Research Council Dyspnea Score, *Adj* Adjusted

## Discussion

The prevalence of abnormal lung functions of any type in this study was 74 % and the prevalence of individual patterns of impairment was 42, 13 and 19 % for obstructive, restrictive and mixed patterns of lung disease respectively. These findings indicate a huge burden of post treatment pulmonary function abnormalities in patients with pulmonary tuberculosis. It was also found that majority of patients with obstructive and restrictive abnormalities had mild to moderate severity. The findings of the present study are similar to those done elsewhere. For instance, in a study by Pasipanodya et al. (2007) in the USA, the prevalence of abnormal lung function of any type was 59 % and the prevalence of individual subtypes of impairment for obstructive, restrictive and mixed were 15, 31 and 13 % respectively [29]. While the prevalence of pulmonary impairment was higher in our study, it suffices to note that pulmonary functions are abnormal in the majority of patients upon completion of chemotherapy. This has been a consistent finding in studies done in other parts of the world [6, 9, 30].

A striking difference can be observed in the proportion of obstructive lung disease between the Pasipanodya study and the present study. The present study revealed a markedly higher proportion of patients who had obstructive lung disease (42 % vs 15 %). In some studies pulmonary infections (like childhood pneumonias, pneumocystis jirovecii, pertussis and measles) contribute up to a third of cases of obstructive airway disease [31–36]. Childhood pneumonias and measles are common in Tanzania. Other studies have found that the odds of developing obstructive disease may be even higher with TB than with smoking [19, 37, 38]. Poor socioeconomic status [39] and low birth weights [40] have also been linked with the development of obstructive impairments both of which are applicable to the Tanzanian setup. For example, 9.7 % of the Tanzanian population are below the food and poverty line [41] and 13 % of Tanzanian newborns are low birth weight [42]. Therefore, a predominance of obstructive sequelae after TB may not be so surprising in this part of the world.

We have shown that patients who presented with recurrent tuberculosis had a 2.8 fold higher likelihood of developing abnormal lung functions at the end of treatment than those with a first episode of TB. Considering that Tanzania is among the high burden countries, the burden of recurrent TB is also high. Studies in other parts of the world have also shown recurrent tuberculosis to be associated with adverse spirometric outcomes. In a population wide Korean study, Lee and colleagues showed that previous TB predicted almost 3 fold higher chance of developing obstructive lung disease [43]. Another South African study of approximately 27,000 miners showed incremental rise in lung function abnormalities with the number of TB episodes (18, 27 and 35 % among first, second and third time sufferers of TB respectively) [8].

In the present study, it was found that HIV negative individuals had a 1.7 fold more likelihood of presenting with any form of lung impairment than were HIV infected patients. These findings can be explained by the fact that the mechanisms by which tuberculosis damages the lung depend extensively on cell mediated immune processes [44]. Many typical tuberculous changes may not be found in individuals who are HIV infected due to defects in their cell mediated immunity. These have been shown in studies comparing radiographic presentations of TB among HIV positive and negative individuals [45]. For example, HIV infected patients may not have typical upper lobe involvement or form typical cavities which confer significant structural alteration to the lung morphology [45]. As a result, it is likely that HIV positive patients end up with less spirometric abnormalities for lack of gross functional alteration due to relatively preserved lung parenchyma unlike their HIV negative counterparts.

The findings of this study warrant an active search among TB patients for these abnormalities. According to these results, practitioners in TB clinics can predict patients who may be at a higher risk for these impairments. Patients with recurrent tuberculosis, patients who are HIV negative, male patients and patients older than 40 years run the highest risk of these abnormalities. The tools to detect such abnormalities are fairly simple, rapid and not overly costly and there should not be a reason to miss this population who may require assistance beyond standard anti tuberculous therapy.

One may argue that these results actually make a case for an even more intensified and rapid detection of TB patients such that earlier treatment may offset the development of these sequelae. While this concept looks plausible in theory, the findings of the present study suggest otherwise. In a subgroup analysis of duration of symptoms to TB diagnosis and lung function, there was no significant difference among patients who were diagnosed and started treatment within 1 month against those who were diagnosed and started treatment later (Table 3). This relationship was also non-significant for obstructive, restrictive and mixed disease suggesting that simply having tuberculosis imparts a risk irrespective of how soon treatment is started.

The prevalence of these abnormalities among patients completing anti TB is alarmingly high. The morbidity and impairment in quality of life conferred by these sequelae is likely to be similarly high. While such data on long term morbidity is missing in Tanzania, literature from other parts of the world suggests that these sequelae confer a significant impact on everyday functioning and quality of life [2, 46–48]. In fact, some studies suggest greater morbidities from the sequelae rather than from the disease itself [2, 48]. These non-communicable post tuberculous sequelae bring to light the often overlooked processes by which TB impacts quality of life. Tuberculosis therefore imposes an infectious and non-infectious burden to the healthcare infrastructure. While the infectious and microbiologic domain has received much attention in TB treatment, a lot is still left to be desired in the non-infectious sequela.

## Conclusions

The magnitude of residual lung function abnormalities among patient with tuberculosis is high despite successful administration of anti TB medications. Important factors associated with abnormal lung functions include recurrent TB, HIV negative status, age more than 40 years and male sex. The findings of this study warrant an active search of these impairments among TB patients. Studies that look at the quality of life and socioeconomic impact of residual lung function abnormalities, screening strategies and treatment need to be conducted to supplement the findings of this study.

### Ethics approval and consent to participate

Ethical clearance for conducting the study was obtained from the Muhimbili University of Health and Allied Sciences (MUHAS) institutional review board.

### Consent for publication

Not applicable.

### Availability of data and materials

The dataset supporting the conclusions of this article are stored with the Directorate of Research and Publications of the Muhimbili University of Health and Allied Sciences and can be made available upon request.

## Abbreviations

Adj: adjusted
ATS: American Thoracic Society
CI: confidence interval
COPD: chronic obstructive pulmonary disease
CRF: clinical record form
DALYS: disability adjusted life years
ERS: European Respiratory Society
FEV1: forced expiratory volume in 1^st^ second
FVC: forced vital capacity
HIV: human immunodeficiency virus
IQR: interquartile range
MMRC: modified medical research council dyspnea score
MUAC: mid upper arm circumference
MUHAS: Muhimbili University of Health and Allied Sciences
OR: odd’s ratio
PFT: pulmonary function test
PTB: pulmonary tuberculosis
SPSS: statistical package for social sciences
TB: tuberculosis
